# Molecular architecture of giant viruses infecting microbial eukaryotes (protists)

**DOI:** 10.5114/bta/208621

**Published:** 2025-09-12

**Authors:** Anhelina Kyrychenko

**Affiliations:** 1 Institute of Evolutionary Biology, Faculty of Biology, University of Warsaw, Poland; 2 D.K. Zabolotny Institute of Microbiology and Virology of the National Academy of Sciences of Ukraine, Kyiv, Ukraine

**Keywords:** *Imitervirales*, metagenomic analysis, nucleocytoplasmic large DNA viruses, protists, virus–host interactions

## Abstract

In this review, I describe recent findings on the molecular architecture and genomic characterization of giant viruses that infect microbial eukaryotes (protists) across diverse ecosystems and ecological niches. Giant viruses are distinguished by their large and complex genomes, which encode a wide range of functions, including protein translation, carbohydrate and lipid metabolism, nitrogen cycling, light assimilation, and key metabolic pathways such as glycolysis and the tricarboxylic acid cycle. Additionally, these genomes feature unique genes, often acquired through horizontal gene transfer, that are not found in other viruses and contribute to the viruses’ ability to manipulate host metabolism and evade host defenses. A core set of genes conserved across different families of giant viruses is highlighted, serving as essential components for key life-cycle processes and providing valuable phylogenetic markers. The review also discusses the role of ORFans and virophages in contributing to the genetic diversity and evolutionary adaptation of these viruses. These findings are crucial for understanding the diversity, evolutionary mechanisms, and complex virus–host interactions of giant viruses, as well as for developing more advanced classification systems. Furthermore, the potential biotechnological applications of unique viral genes and pathways are explored, underscoring the importance of ongoing research in this field.

## Introduction

**Figure 1:**
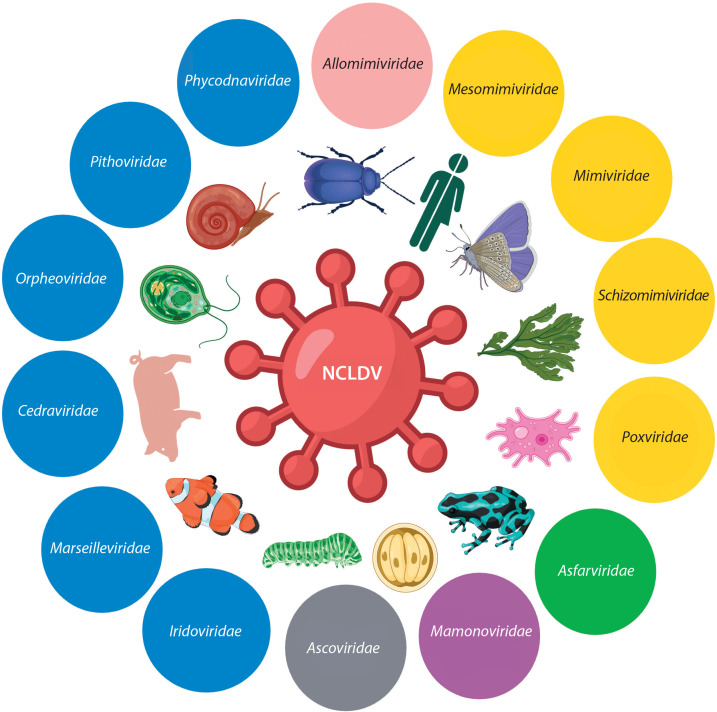
Schematic representation of nucleocytoplasmic large DNA viruses families, illustrating the diversity of giant viruses. Created in https://BioRender.com

Nucleocytoplasmic large DNA viruses (NCLDVs) are large double-stranded DNA eukaryotic viruses that infect a variety of hosts, including protozoa, invertebrates, and eukaryotic algae (Figure 1). Viruses in this monophyletic group — also known as giant viruses — replicate either fully or partially in the cytoplasm of eukaryotic cells and are characterized by huge genomes and virion sizes, which can reach 2.5 Mb and 1.5 μm, respectively. The discovery of Acanthamoeba polyphaga mimivirus (APMV) in amoebae in 2003 surprised the scientific community due to its enormous genome size, unprecedented genetic complexity, and the presence of genes previously thought to be exclusive to cellular life, including those related to translation, DNA repair, and chaperone production (La Scola et al. 2003; Raoult et al. 2004). This discovery spurred more active searches for giant viruses in diverse environments, leading to a deeper understanding of their genome organization, replication mechanisms, transcriptional processes, virulence factors, genetic diversity, and phylogenetic relationships. Moreover, the discovery of giant viruses has challenged earlier assumptions about the structural and genomic simplicity of viruses and has led to a refinement of classical virus demarcation criteria. This has prompted renewed interest in viruses, their evolution, and their potential roles in ecosystems (Bosmon et al. 2025).

**Figure 2:**
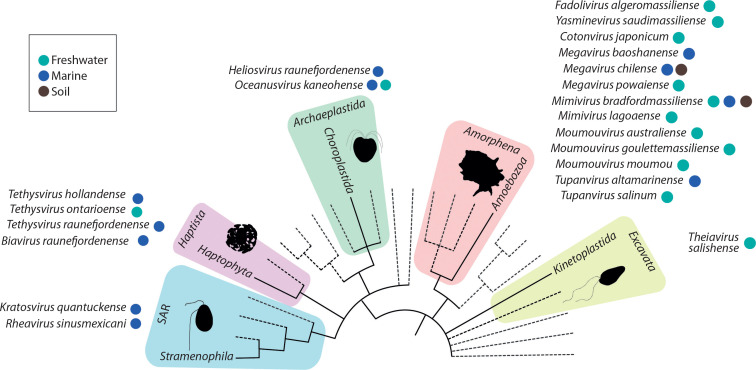
Giant viruses found in major protist lineages. Created in https://BioRender.com

Giant viruses are primarily known to infect protists, a diverse group of eukaryotic microorganisms that includes algae, amoebae, ciliates, and other single-celled organisms (Fischer 2016; Adl et al. 2018; Kostygov et al. 2021; Queiroz et al. 2024). These viruses have been identified across various protist clades, reflecting their broad distribution and diverse host range (Figure 2). Protists play crucial roles in aquatic and terrestrial ecosystems, contributing to vital processes such as nutrient and carbon cycling, primary production, and energy flow. They inhabit a range of environments — including freshwater and marine systems, terrestrial habitats, and symbiotic or parasitic relationships within other organisms — and have even been discovered in extreme habitats (Rappaport and Oliverio 2023). The ubiquity and ecological significance of protists make them valuable model systems for in-depth studies of cell biology and for understanding complex cellular processes, functions, and responses to viral infections at the molecular and structural levels (Queiroz et al. 2024).

Understanding the prevalence and diversity of giant viruses infecting protists is essential for several reasons. First, it sheds light on their impact on ecosystems and their potential roles in protist biology and evolution, as giant viruses can significantly alter protist population dynamics through widespread genetic and metabolic reprogramming (Hurwitz et al. 2013; Ku 2021; Queiroz et al. 2024). Second, studying NCLDV genomes helps elucidate the mechanisms of virus evolution and diversification, revealing how these entities have acquired complex metabolic capabilities, developed sophisticated functional features, and adapted to their hosts (Schulz et al. 2022; Tee and Ku 2025). Furthermore, the impact of giant viruses on human health remains unclear and represents an emerging area of research. Current data on the identification of viral sequences in the human gut microbiome, the ability of some giant viruses to replicate in human peripheral blood cells, and their capacity to activate the interferon system suggest potential implications. However, many aspects of the giant virus–human relationship — such as pathogenicity, transmission dynamics, and overall influence on human health — remain to be elucidated (Brandes and Linial 2019; Athira and Antony 2023). Additionally, the unique biochemical pathways and enzymes found in giant viruses offer potential applications in biotechnology and medicine, as their complex enzymatic machinery presents new opportunities for innovative uses (Brahim Belhaouari et al. 2022; de Oliveira et al. 2022).

Recent taxonomic updates classify NCLDVs within the phylum *Nucleocytoviricota*, which encompasses several approved viral orders (*Algalvirales, Asfuvirales, Chitovirales, Imitevirales*, and *Pimascovirales*, plus one unassigned order) and 11 taxonomic families (Figure 1) (Aylward et al. 2023; ICTV 2025). Viruses within this phylum possess large double-stranded DNA (dsDNA) genomes, replicate partially or entirely in the host cytoplasm, and share genetic similarities that support a hypothesized common origin. The latest classifications are based on comprehensive analyses of gene content, morphology, and phylogenomics, with a strong emphasis on phylogenetic relationships and an evolutionary framework (Koonin et al. 2020; Simmonds et al. 2023).

Giant viruses have highly complex and chimeric genomes that reflect dynamic gene exchange with their host cells and other viruses. In addition to the basic machinery for virion structure and DNA replication, these viruses often encode genes involved in translation, glycolysis, the tricarboxylic acid cycle, cytoskeletal dynamics, light harvesting, nutrient transport, and other pathways essential for nutrient homeostasis (Iyer et al. 2006; Endo et al. 2020; Farzad et al. 2022; Schulz et al. 2022). These findings collectively suggest that giant viruses employ a diverse array of functional genes to manipulate host physiology and modify the intracellular environment to facilitate virion propagation.

Recent advances in metagenomic techniques, bioinformatics tools, and high-throughput sequencing technologies have enabled the discovery of previously unidentified NCLDVs from environmental samples in a cultivation-independent manner (Tang 2020). These tools allow for the reconstruction of individual NCLDV genome sequences from metagenomic datasets and the exploration of predicted functions encoded in their genomes (Tang 2020; Farzad et al. 2022; Schulz et al. 2022). By applying sequence-based homology searches, computational predictions from bioinformatic analyses, domain and motif analyses, and functional annotations, researchers can discover, identify, and classify giant virus genes and proteins, assess their diversity and functional potential, and better understand how they influence host metabolism and drive cellular reprogramming.

This review presents current data on the genomic organization of giant viruses infecting protists, with a focus on the functional features of newly identified genes in NCLDV genomes and the roles they may play in virus–host interactions. Giant viruses infect a broad spectrum of eukaryotic hosts; members of the *Asfuvirales, Chitovirales*, and *Pimascovirales* orders infect various metazoan and protist hosts, while viruses within the *Algavirales* and *Imitervirales* orders typically infect algae, amoebae, and other protists (Weynberg et al. 2017; Wilhelm et al. 2017; Claverie and Abergel 2018; Karki et al. 2021; Koonin and Yutin 2019; Ha and Aylward 2024). The present discussion is limited to viruses from the *Imitervirales* order, as they are especially abundant and widely distributed across diverse environments. These viruses possess complex gene structures, and their genomes contain genes with a wide range of functions, including light-sensitive proteins (rhodopsins), structural proteins of the cytoskeleton, and enzymes involved in central metabolic pathways such as the TCA cycle and glycolysis (Abrahăo et al. 2018).

Recent changes in virus taxonomy — particularly the recognition of *Allomimiviridae, Mesomimiviridae*, and *Schizomimiviridae* as distinct families within the *Imitervirales* — have expanded our understanding of the diversity among viruses closely related to *Mimivirus*, often described as the “extended *Mimiviridae*” group (Aylward et al. 2023; ICTV 2025). This overview may be useful for researchers studying viral evolution and virus–host interactions, as well as for molecular biologists and biotechnologists exploring the potential applications of enzymes encoded by giant viruses.

### Genes and proteins of Imitervirales

#### General genome architecture of Mimiviridae

The *Mimiviridae* is one of the largest and most diverse families of eukaryotic viruses. It includes unusually large viruses that infect a range of protists, particularly amoebae. These viruses are notable for their exceptionally large virion sizes (> 400 nm) and enormous genomes, which encode more than 900 proteins. *Mimivirus*, a prominent member and the prototype of the *Mimiviridae* family, carries a linear double-stranded DNA genome of approximately 1.2 Mbp — exceeding the genome sizes of most known viruses and even some bacteria, archaea, and lower eukaryotes (Abrahăo et al. 2018; Koonin et al. 2015). More than 60% of the genes found in *Mimiviridae* viruses have no currently assigned function. The remaining genes are primarily involved in processes essential for viral survival, such as nucleotide synthesis, recombination, DNA repair, protein translation, and other pathways typically found in cellular organisms (Claverie and Abergel 2018; Dos Santos Silva et al. 2020). Given their genome size and unexpected complexity, these viruses have drawn considerable scientific interest, particularly in studies of their molecular organization and the functions of the proteins they encode.

The gene and protein arsenal of *Mimiviridae* can be categorized into several functional groups: structural proteins (e.g., capsid proteins, membrane proteins); enzymes involved in replication and transcription (e.g., DNA polymerases, helicases); translation components (e.g., aminoacyl-tRNA synthetases); host interaction and modulation proteins (involved in host defense evasion and manipulation); metabolic enzymes (e.g., those involved in nucleotide and lipid metabolism); and accessory proteins, such as protein kinases and ubiquitin-related factors (Claverie et al. 2009). Unique to these viruses are genes canonically involved in signal transduction (e.g., serine/threonine kinases and phosphoinositide 3-kinase), DNA processing, and assembly, as well as virally encoded DNA repair systems like base excision repair (BER) enzymes (Claverie et al. 2009; Colson et al. 2018; Kalafati et al. 2022; Lad et al. 2023).

In addition to the core gene sets common to NCLDVs, mimiviruses possess several unexpected genes, such as components of the translation system that are unable to initiate transcription of early genes in the host cytoplasm immediately after infection (Kalafati et al. 2022). Notably, *Mimivirus* and related viruses carry genes involved in DNA repair, including those encoding a mismatch-recognition protein (MutS) homolog, NEIL-1 and NEIL-2 proteins, and PrimPol – enzymes that function within the BER pathway. The *Mimivirus* polymerase X protein (mvPolX) also contributes to the repair process by binding gapped DNA substrates and performing single-nucleotide gap filling, followed by downstream strand displacement (Lad et al. 2023). These genes suggest a high degree of replication autonomy and the presence of complex genome maintenance mechanisms (Bandaru et al. 2007; Lad et al. 2023).

In addition, the presence of histone-like proteins (homologs of eukaryotic histones) encoded in the viral genome and involved in organizing genomic DNA within the capsid — found in *Mimiviridae* and certain other viruses of the NCLDV group — represents a unique feature that reflects the complex evolutionary history of these viruses and their possible connections to early eukaryotic hosts (Sharma et al. 2016; Talbert et al. 2022; Sharma et al. 2024). Genome analyses of these viruses suggest that many viral genes were acquired through horizontal gene transfer from their hosts, coinfecting viruses, or other intracellular parasites existing within the same cell (Kalafati et al. 2022).

*Mimiviridae* genomes also exhibit high proportions of duplicated genes, ORFans (genes with no known homologs), and transpovirons — mobile genetic elements resembling transposons (Colson et al. 2018; Dos Santos Silva et al. 2020; Kalafati et al. 2022). These features contribute to the unique structural and functional properties of the viruses, possibly aiding in host interactions and adaptation. Furthermore, the presence of sequences resembling intronic regions of 18S ribosomal RNA genes further underscores the complexity of the viral genome (Abrahăo et al. 2018). While the exact functional roles of these intronic sequences remain under investigation, their presence suggests potential regulatory functions in viral gene expression or interference with host cellular processes.

The large genomes of giant viruses, along with multiple packaged proteins, require genome condensation. In viruses belonging to the phylum *Nucleocytoviricota*, this condensation is believed to be facilitated by histone-like or nonhistone proteins. The discovery of proteins such as gp275 — an architectural protein involved in organizing genomic DNA within the *Mimivirus* capsid and similar to the histone-like methanogen chromosomal protein 1 (MC1) — highlights the sophisticated mechanisms these viruses employ for genome condensation and protection. Remarkably, no DNA-condensing protein responsible for assembling the enormous genome had been reported until this discovery (Sharma et al. 2024).

Another notable feature of the *Mimiviridae* is the presence of cyclophilins (CYNs), prolyl isomerases not typically found in viruses (Barik 2018). CYNs are known for their peptidyl-prolyl cis-trans isomerase activity and important roles in protein folding and chaperone function. Viral CYNs may participate in viral replication and host cell manipulation, indicating a complex interplay between virus and host. The origin of giant virus CYNs remains unclear and is hypothesized to involve lateral gene transfer among mimiviruses, potentially reflecting adaptation to the specific needs of individual viruses (Barik 2018). The study of viral CYNs is a growing field that promises to deepen our understanding of evolutionary mechanisms, functional specificity, and their potential roles in the life cycle of giant viruses and host–virus interactions.

**Figure 3:**
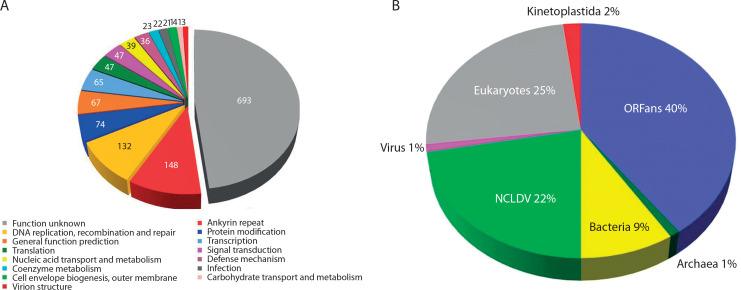
BsV genome content: **A**) Functional assignment of BsV genome content. **B**) Domain of best BLASTp hits (adapted from Deeg et al. 2018)

An example of the surprising genomic plasticity of giant viruses is *Theiavirus salishense* (formerly Bodo saltans virus, BsV), which possesses one of the largest sequenced genomes within the *Mimiviridae* family and is among the most abundant members of this family in aquatic ecosystems (Figure 3) (Deeg et al. 2018). Its 1.39 Mb genome encodes 1,227 predicted ORFs, including complex replication machinery. Notably, BsV has lost much of its translational apparatus, including all tRNAs. Essential genes are invaded by homing endonuclease – encoding self-splicing introns, which may function as a defense mechanism against competing viruses. The presence of putative antihost factors, accompanied by extensive gene duplication via a “genomic accordion” mechanism, suggests an ongoing evolutionary arms race. This rapid evolution and genomic plasticity have contributed to the phenomenon of genome gigantism and highlight the unique nature of giant viruses (Deeg et al. 2018).

### General genome architecture of Allomimiviridae

The *Allomimiviridae* family, part of the recently classified *Imitervirales* order, shares many characteristics with other giant viruses, particularly those in the *Mimiviridae* family. This family includes species such as *Heliosvirus raunefjordenense*, formerly known as Pyramimonas orientalis virus 01b (PoV-01b), and the recently cultivated *Oceanusvirus kaneohense*, formerly known as Tetraselmis virus (TetV). Both are marine viruses that infect green algae – specifically *P. orientalis* and *Tetraselmis* sp., respectively (Aylward et al. 2023).

The genomic structure of *Allomimiviridae* is consistent with other mimiviruses in terms of gene content and organization. It is complex and contains a wide array of functional genes. For instance, PoV-01b has a genome size of 560 kbp and encodes structural proteins, DNA and RNA polymerases, helicases, tRNA synthetases, and proteins involved in host interactions. The virus particles measure approximately 220 × 180 nm, with the major polypeptide being 44 kDa in size (Sandaa et al. 2001).

Among the notable genome features of viruses infecting *P. orientalis* are the channelrhodopsin (ChR) genes. These genes, homologous to those found in the host algae and other organisms, suggest recent acquisition from a close relative within the prasinophyte green algal classes: *Pyramimonadophyceae, Mamiellophyceae*, and *Nephroselmidophyceae* (Gallot-Lavallée and Archibald 2020; Rozenberg et al. 2020). These viral ChRs, identified through bioinformatic analysis of microbial metagenomic data and their algal homologs, act as anion-conducting channels. They may enhance viral replication and spread by manipulating the host’s cellular processes, such as membrane potential and ion flow, potentially affecting the host’s swimming behavior (Rozenberg et al. 2020). The growing number of viral rhodopsins identified among NCLDVs raises questions about their role during infection, including whether they modify host phototaxis or disrupt membrane potential – possibly leading to cell death (Oppermann et al. 2023).

Although the structure, function, and role of viral rhodopsins in host protist infection remain unclear, the distinct functional properties of these ChRs — such as their light activation spectra and ion selectivity — present intriguing targets for further biochemical and biophysical studies. The presence of ChR genes in *P. orientalis* viruses highlights how viruses can acquire and potentially repurpose host genes to manipulate cellular processes. These viral ChRs not only provide insights into virus–host interactions but also hold promise for developing new tools in optogenetics. Further research into their functional properties and potential applications could deepen our understanding of their roles in viral biology and their technological uses.

Tetraselmis virus 1 (TetV) infects the cosmopolitan green alga *Tetraselmis* (*Chlorodendrophyceae*), with virions measuring on average between 226 ± 9 and 257 ± 9 nm. Its circular dsDNA genome spans 668 kb and contains 663 predicted genes, including 653 coding DNA sequences (CDSs) and 10 tRNA genes within an 879-bp region (Schvarcz and Steward 2018). In addition to genes involved in replication, recombination, transcription, and translation, TetV encodes six serine/threonine protein kinases associated with post-translational modification and signal transduction (Schvarcz and Steward 2018). The transcription-related and translation-related genes are highly similar to those found in other algal-infecting mimiviruses. The genome also features a functional potassium (K^+^) channel — similar to other representatives of *Nucleocytoviricota* — which may confer an advantage during infection of algal hosts (Kukovetz et al. 2020).

Many of TetV’s predicted proteins are novel, showing no similarity to known proteins, and include a significant number of eukaryote-derived proteins as well as proteins similar to those found in viruses and bacteria (Schvarcz and Steward 2018). Of the 653 predicted protein-coding genes in the TetV genome, only 192 have been functionally annotated. Among these are genes encoding two key fermentation enzymes — pyruvate formate-lyase and pyruvate formate-lyase activating enzyme — proteins with unique functions not previously identified in viruses. These enzymes are involved in cellular fermentation pathways that enable energy production in the absence of oxygen. This is particularly relevant because *Tetraselmis*, the only known host of TetV, utilizes anaerobic energy metabolism under low-oxygen conditions, suggesting a possible case of horizontal gene transfer (HGT) (Brahim Belhaouari et al. 2022). Alternatively, this gene distribution may reflect differential gene loss in other eukaryotic lineages (Ku et al. 2015; Bremer et al. 2025). Notably, *Tetraselmis* and other green algae possess some of the most complete, vertically inherited sets of genes for anaerobic energy metabolism among eukaryotes (Gould et al. 2019), suggesting that these genes may have been retained rather than acquired. Furthermore, the presence of these enzymes implies that TetV can manipulate the fermentation pathway of infected algal cells, alter host metabolic dynamics, and enhance viral replication and spread – potentially leading to significant ecological consequences.

Other unique genes identified include those encoding a-galactosidase and mannitol 1-phosphate dehydrogenase (M1PHD), which are involved in saccharide degradation and mannitol metabolism, respectively (Schvarcz and Steward 2018). Additionally, TetV possesses genes related to ubiquitination, methylation, DNA repair, and a unique eukaryotic initiation factor, eIF-1A, not observed in other algal-infecting mimiviruses (Iyer et al. 2006; Schvarcz and Steward 2018).

Overall, the genomes of *Allomimiviridae*, similar to those of other giant viruses in the phylum *Nucleocytoviricota*, are mosaic in nature and contain genes from a variety of sources — including other viruses, bacteria, archaea, and eukaryotes. This evidence of HGT underscores a long history of co-evolution between these viruses and their eukaryotic hosts, contributing to the large and diverse gene repertoires seen in giant viruses.

### General genome architecture of Mesomimiviridae

*Mesomimiviridae*, a recently recognized group within the order *Imitervirales*, is particularly prevalent in aquatic systems (Aylward et al. 2023; Farzad et al. 2022). This family includes viruses such as *Tethysvirus hollandense* (formerly Phaeocystis globosa virus isolates, PgV), *Tethysvirus ontarioense* (formerly Chrysochromulina parva virus BQ2, CpV-BQ2), and *Tethysvirus raunefjordenense* (formerly Chrysochromulina ericina virus CeV-01B, CeV), which infect haptophytes — a group of unicellular algae (Aylward et al. 2023).

The genomes of *Mesomimiviridae* are characterized by their moderate size (~500 kb) compared to other members of *Imitervirales*. Approximately 70% of the linear dsDNA genome of PgV is conserved with other large dsDNA viruses and includes core genes essential for viral replication and repair, such as methyltransferases and transposases (de Oliveira et al. 2022). These genomes also encode capsid proteins and other structural components necessary for virus assembly. Of the 434 putative CDSs identified in the PgV genome, only 150 (34.6%) have predicted functions based on homology — reflecting the typical proportion of uncharacterized genes in large eukaryotic viruses. Unique enzymes, such as phospholipase and asparagine synthetase homologs, are also encoded within these genomes (Santini et al. 2013).

Genes encoding proteorhodopsin, identified in the genomes of PgV, provide another example of viral manipulation of host primary metabolism. The origins of viral proteorhodopsin genes remain unknown, but they may have been captured from their unicellular eukaryotic hosts (Kukovetz et al. 2020). In addition to DNA-manipulating enzymes common to most giant viruses, PgV possesses DNA repair enzymes such as an apurinic/apyrimidinic exonuclease and a mismatch repair enzyme, both involved in correcting errors that occur during DNA replication (Endo et al. 2020; Santini et al. 2013). The PgV genome is also rich in putative ribonucleases, including ribonuclease HII, an RNase T-like enzyme, and a PIN-domain-containing RNase H. These enzymes contribute to the removal of RNA primers during DNA replication, the repair of RNA–DNA hybrids, and possibly RNA processing and degradation. The specific role of the asparagine synthase genes identified in *Phaeocystis globosa* virus during infection remains unclear (Farzad et al. 2022).

The remaining members of *Mesomimiviridae*, such as *Tethysvirus ontarioense* (CpV-BQ2) and *Tethysvirus raunefjordenense* (CeV) — marine viruses infecting *Chrysochromulina* species — share similarly large dsDNA genomes and complex gene content but differ in their specific host ranges. CeV, for example, has a 474-kb genome containing 512 putative protein-coding genes and 12 tRNA genes (Gallot-Lavallée et al. 2017; Kyrychenko et al. 2023). Many of these proteins have no significant matches in the NCBI nonredundant sequence database, with the majority of identified homologs being viral. Among the 293 predicted proteins with database homologs, 75.4% matched best to eukaryote-infecting large dsDNA viruses. The best nonviral matches were distributed among bacteria, eukaryotes, and host haptophytes, suggesting possible HGT and the inheritance of genes from common ancestors of eukaryote-infecting large dsDNA viruses (Kyrychenko et al. 2023). Among the 305 proteins unique to CeV (i.e., without recognizable homologs in the other *Mimiviridae*, including 68% ORFans), only 106 (35%) have a database homolog, of which only 52 are associated with functional attributes. Unique features of the CeV genome include the presence of DNA repair nucleases, such as ERCC4, useful for repairing UV-induced genome damage (Gallot-Lavallée et al. 2017).

CeV and PgV share homologs of the cold shock protein, which is known to act as an RNA chaperone. Additionally, CeV encodes inteins — mobile genetic elements that function as protein introns, removing themselves from host proteins through autocatalytic excision and protein splicing. It is unusual not only that these genes are present in the viral genome, but also that there are so many (eight inteins), which are strongly biased toward DNA-processing enzymes (Gallot-Lavallée et al. 2017). The presence of unique elements such as ERCC4, a DNA repair nuclease, and multiple inteins involved in protein splicing highlights the complexity and distinct adaptations of these viruses.

CpV-BQ2, like other members of the *Mesomimiviridae* family, possesses a mosaic gene composition derived from various sources — including viruses, bacteria, and eukaryotes. As with other giant viruses, a significant fraction of its genes have no readily detectable homologs. The 437-kb genome encodes 503 ORFs, which include typical NCLDV proteins involved in core viral functions, as well as unique gene clusters such as those encoding E3 ubiquitin ligases. These ligases may inhibit host defense mechanisms, highlighting the virus’s complex strategies for evading host immunity. Additionally, CpV-BQ2’s gene repertoire includes DNA and chromatin modification enzymes, such as DNA methyltransferases and histone modification enzymes, which likely target host chromatin (Stough et al. 2019).

In addition to a conserved set of genes common to NCLDVs, the CpV-BQ2 genome contains genes involved in central carbon metabolism and light harvesting. Notably, the CpV genome hosts the genomes of three distinct virophages, which co-occur with the virus and likely exploit CpV for their replication (Tokarz-Deptuła et al. 2023). These virophages encode a variety of genes, including both functionally characterized proteins and others with uncharacterized or unexpected functions. The identification of chlorophyll-binding proteins and genes related to the TCA cycle in *Mesomimiviridae* members suggests that these viruses may manipulate host metabolic pathways, particularly those involved in energy production and photosynthesis (Farzad et al. 2022).

For a detailed description of the predicted genes of viruses infecting *T. hollandense* and *Chrysochromulina* species (haptophytes), refer to the NCLDV metagenome-assembled genomes (MAGs) list, which compiles data from environmental metagenomic datasets collected globally. These MAGs provide the best matches to transcripts of chrysophytes, further elucidating the genomic complexity and diversity within the *Mesomimiviridae* family (Endo et al. 2020).

Like other viruses in the order *Imitervirales*, members of the *Mesomimiviridae* family generally possess genomes with complex and diverse gene content. In addition to their primary functions, these viruses contain genes encoding structural proteins and enzymes involved in amino acid metabolism, DNA processing, central carbon metabolism, and light harvesting. These features suggest that *Mesomimiviridae* may employ various strategies to manipulate host cellular physiology during infection (Farzad et al. 2022).

### General genome architecture of Schizomimiviridae

The *Schizomimiviridae* family, previously referred to as the *Aureococcusvirus* group, includes two representative species: *Biavirus raunefjordenense* and *Kratosvirus quantuckense*, formerly known as Prymnesium kappa virus RF01 (PkV-RF01) and Aureococcus anophagefferens virus BtV-01 (AaV), respectively (Aylward et al. 2023). These marine giant viruses infect haptophyte and heterokont hosts and exhibit genomic features and host interactions that distinguish them from other members of *Imitervirales*. The presence of hallmark genes such as DNA polymerase B (PolB) and the major capsid protein (MCP) places these viruses within the NCLDV group and reflects their conserved roles in replication and virion structure (Yutin and Koonin 2012; Johannessen et al. 2014).

*Biavirus raunefjordenense* (PkV-RF01) has a linear DNA genome of approximately 1.42 Mb, comprising 1,161 genes, including 1,121 coding sequences and 40 tRNA genes. Notably, PkV-RF01 contains a complete base excision repair (BER) pathway — a feature also observed in some *Mimiviridae* infecting heterotrophs — suggesting enhanced capabilities for DNA repair and protein synthesis (Blanc-Mathieu et al. 2021).

PkV-RF01 is distinguished by an extensive array of genes involved in energy metabolism, including those associated with the tricarboxylic acid (TCA) cycle and the β-oxidation pathway (Blanc-Mathieu et al. 2021). Among these, the gene encoding succinate dehydrogenase subunit A is actively transcribed during infection, highlighting the virus’s potential to regulate host energy metabolism. Furthermore, PkV-RF01 encodes a substantial number of glycosyltransferases and other carbohydrate-active enzymes — surpassing the numbers found in many other viruses (Blanc-Mathieu et al. 2021).

Initially identified as Aureococcus anophagefferens virus (AaV), *Kratosvirus quantuckense* was isolated in 2002 and sequenced twice — in 2014 and 2022 — to better unravel its functionality by determining its genetic potential (Truchon et al. 2023). According to the first sequencing, *Kratosvirus quantuckense* has a relatively small dsDNA genome of approximately 370.92 kb, encoding 377 putative coding sequences and exhibiting a genome coding density of 88.3%, typical of large dsDNA viruses. The genome harbors NCLDV-specific core genes and a substantial proportion (71.35%) of unique genes, many of which appear to have been acquired horizontally from the host and other sources. These include genes involved in DNA replication, transcription, and translation (Truchon et al. 2023).

AaV, along with two other photosynthetic protozoan viruses – Phaeocystis globosa virus (PgV) and Chrysochromulina ericina virus (CeV) – shares a small set of seven genes that distinguish them from other members of the *Mimiviridae* family. Among these shared genes, only one has a predicted function: an ERCC4-type DNA repair nuclease. This enzyme is typically associated with the cellular response to UV-induced DNA damage, suggesting a potential role in protecting the viral genome from such damage. The presence of this specific gene among these viruses may indicate an evolutionary adaptation to environments with high UV exposure (Gallot-Lavallée et al. 2017).

The limited number of shared genes among AaV, PgV, and CeV – despite their common infection of photosynthetic protozoan hosts – suggests that their genomic features are not primarily dictated by lifestyle or host type. This points to a significant degree of genomic diversity and specialization within these viruses, possibly driven by factors such as ecological niche, evolutionary history, or specific host–virus interactions.

According to genomic sequencing results from 2022, the AaV genome is 381,717 bp long, with 384 CDSs and an additional 5-kb region located between the previously predicted polar ends of the reference genome (Truchon et al. 2022). Other observed changes include apparent duplications, gene elongations, and gene fusions missed during the initial assembly (Truchon et al. 2022). A notable feature of the AaV genome is the presence of two subunits of DNA-directed RNA polymerase 2 (Rpb2), likely resulting from an ancient gene duplication event. This duplication may contribute to the virus’s functional diversification and adaptation (Truchon et al. 2022). Although the role and frequency of gene duplication events remain unclear, their occurrence suggests functional significance that may enhance viral fitness (Truchon et al. 2023). Overall, the genomic organization of AaV appears to reflect genome expansion driven by gene duplication and the acquisition of host-derived genes (Truchon et al. 2022, 2023).

AaV also contains unique genes for pectate lyases — typically involved in plant tissue degradation — and concanavalin A-like lectin/glucanase genes, suggesting roles in carbohydrate metabolism during infection (Truchon et al. 2022, 2023). These nonglycosyltransferase carbohydrate metabolism genes are hypothesized to play important roles during infection. The packaging of one pectate lyase and three lectin/glucanases within the viral particle underscores their importance, particularly for metabolic processes initiated immediately upon host invasion (Truchon et al. 2022, 2023). Additionally, AaV includes a viral tRNATyr gene with a 20 bp intron in the canonical position between nucleotides 37 and 38 of the tRNA precursor — a characteristic shared with most bacterial, plastid, eukaryotic, and archaeal tRNAintronic RNAs (Bhattacharjee et al. 2023).

The AaV genome is characterized by a significantly lower GC content (28.7%) compared to its host’s (69.9%), suggesting adaptations that support nucleotide salvage pathways (Truchon et al. 2023). Moreover, compared to other *Nucleocytoviricota*, the virus encodes an unusually large number of methyltransferases — eight, according to the initial annotation of *Kratosvirus quantuckense* — which may function in mRNA capping and DNA methylation, highlighting the complexity of its host–virus regulatory mechanisms (Truchon et al. 2022). Understanding the specific genetic traits and functional capabilities of *K. quantuckense* is essential for developing innovative molecular tools and bioinformatics methods to define similar uncultured viruses and study viruses across diverse environments.

## Conclusions

The genomes of giant viruses are remarkably diverse, encompassing genes responsible for a wide array of functions, including protein translation, carbohydrate and lipid metabolism, nitrogen cycling, and light assimilation. These capabilities suggest that giant viruses can significantly alter the metabolic processes of their hosts, affecting pathways such as glycolysis, gluconeogenesis, and the tricarboxylic acid cycle. Such modifications highlight the complex interactions these viruses have with their host organisms.

Table 1 presents the main characteristics of NCLDVs infecting protists, including genome size, capsid properties, host range, and unique viral genes. A core set of genes, conserved across various families of giant viruses, encodes essential proteins for key life-cycle processes. The selection of markers used to assign a virus to the NCLDV group and classify it within the group remains an active area of research. In addition to the diverse set of genes used to determine evolutionary relationships, researchers employ different analytical strategies. The use of single-gene markers – such as the major capsid protein or DNA polymerase – provides only moderate resolution, particularly at higher taxonomic levels, and may be affected by horizontal gene transfer or recombination events (Larsen et al. 2008; Chen and Suttle 1996; Rowe et al. 2011).

**Table 1:** Basic characteristics of NCLDVs infecting protists

Family	Genome size (kb)	Capsid: symmetry/size (nm)/features	Natural or experimental host	Unique genes involved in (related to)
*Allomimiviridae*	500–600	Ico/220–260/glycosylated fibrils, protrusions	Chlorophyta	host interaction and metabolism, fermentation, anion-conducting channelrhodopsins
*Mimiviridae*	1020–1260	Ico/300–500/heavily glycosylated fibrils	Amoebae SAR supergroup Kinetoplastida Stramenopiles	translation, DNA repair, carbohydrate metabolism
*Schizomimiviridae*	370–1420	Ico/250–300/long surface fibrils	Haptophyta SAR supergroup Pelagophytes Heterokonts	glycosyltransferases, enzymes for energy metabolism
*Mesomimiviridae*	430–600	Ico/300–400/glycoproteins, fibrils	Haptophyta (*Chrysochromulina*)	DNA and chromatin modification enzymes, energy metabolism
*Phycodnaviridae*	160–560	Ico/130–150/lipid envelope in some members	Alveolata Chlorophyta Haptophyta Stramenopiles	photosynthesis, glycosyl-transferases, dinoflagellate/viral nucleoproteins, host cell apoptosis, signaling pathways, transcription, DNA polymerase
*Mamonoviridae*	360–382	Ico/~260/surface spikes	Amoebae (*Acanthamoeba*)	full set of histone proteins, lack two of the core genes – RNA polymerase and DNA topoisomerase II
*Marseilleviridae*	300–400	Ico/200–400/surface protrusions and glycoproteins	Amoebae (*Acanthamoeba*)	cell signaling, metabolism
*Cedraviridae*	460–600	Ico/150–200/surface proteins, two apex corks	Amoebae (*Acanthamoeba*)	DNA replication, repair
*Orpheoviridae*	1474	Ico/900–1300/ostiole at the apex	Amoebae (*Acanthamoeba*)	metabolic and regulatory genes
*Pithoviridae*	600–700	Ico/1500/fibers, single apex cork projections	Amoebae (*Acanthamoeba*)	transcription and translation

*Ico – icosahedral symmetry

In contrast, multigene approaches using sets of 5–50 conserved genes involved in major viral functions provide higher phylogenetic resolution, especially for reconstructing deep evolutionary splits and preserving the monophyly of major NCLDV clades (Koonin and Yutin 2010; Aylward et al. 2021; Sun and Ku 2021). Among these conserved markers, genes such as *PolB* (DNA polymerase family B), *RNAPL* (RNA polymerase large subunit), A32-like packaging ATPase, topoisomerase II, *VLTF3, TFIIB*, and superfamily II helicase are frequently used due to their length, conservation, and functional significance. Some studies have also employed whole-proteome approaches, which offer high phylogenetic resolution but require complete or nearcomplete genome sequences (Yu et al. 2010).

A subgroup of ten highly conserved key genes (phylogenetic markers) has been proposed as a reliable phylogenetic framework for comparative analysis, lineage classification, and evolutionary studies of NCLDVs. These include the A2L-like transcription factor, A32-like packaging ATPase, D5-like helicase-primase, elongation subunit of B-family DNA polymerase, helicase II, large subunit of the mRNA capping enzyme, myristoylated envelope protein, small subunit of ribonucleotide reductase, and the α-subunit and β-subunit of RNA polymerase (Athira and Antony 2023; Campillo-Balderas et al. 2023).

Beyond conserved core genes and established phylogenetic markers, other genomic elements – such as ORFans and virophages – further contribute to the diversity and evolutionary potential of NCLDVs. ORFans – genes without known homologs in viruses infecting unicellular eukaryotes, particularly within the NCLDV group – add to the functional complexity and unique characteristics of these viruses. Virophages, by integrating into giant virus genomes, can influence viral functionality through mechanisms such as horizontal gene transfer, modulation of viral replication, and alteration of host–virus dynamics. These interactions can drive viral evolution, contributing to the complexity and adaptability of giant viruses.

Recent studies show that gene expression in giant viruses follows a well-regulated temporal pattern and occurs in three phases: early (DNA replication, host invasion), intermediate (nucleotide metabolism), and late (morphogenesis and structural protein synthesis) (Moniruzzaman et al. 2018; Bessenay et al. 2024; Chen et al. 2024). In each phase, the virus activates specific groups of genes that control different steps of the infection cycle: early genes initiate DNA replication and host invasion, intermediate genes support nucleotide synthesis, and late genes control proper virion assembly. This stepwise expression program illustrates the complex interplay between the virus and host machinery, as well as the virus’s ability to manipulate host processes throughout infection (Moniruzzaman et al. 2018; Chen et al. 2025).

During the infectious cycle, the virus not only reprograms the expression of its own genes but also induces large-scale changes in the host cell transcriptome. According to Nuri et al. (2022), *Mimivirus* infection in *Acanthamoeba polyphaga* triggers cell cycle arrest and complex intracellular modifications that support viral replication. In addition to interfering with cell cycle progression and redirecting host metabolic pathways, the virus induces morphological changes – such as cell rounding and loss of pseudopodia – by downregulating cytoskeleton-related genes and upregulating genes involved in secretory pathways, peroxisomes, and the ubiquitin–proteasome system. These changes facilitate viral replication and may contribute to viral factory formation through translocation of endoplasmic reticulum membranes, reorganization of the cytoskeletal network, and sustained cell cycle arrest.

This already complex expression program may be further influenced by additional factors. In the Megamimivirinae subfamily, for instance, transcription is modulated by hyperparasitic virophage co-infections and transpovirons – plasmid-like linear dsDNA molecules – all of which share common transcriptional regulatory elements (promoter motifs) (Ku et al. 2020; Bessenay et al. 2024; Bosmon et al. 2025). Interestingly, virophage co-infection transiently alters giant virus gene expression, while transpovirons appear to play a commensal role with no significant impact, suggesting a rich network of interactions. These close transcriptional–level interactions affect each partner in this complex relationship and influence specific cellular functions.

Moreover, environmental factors such as nutrient availability also influence viral transcription, adding another layer to the complexity of gene regulation (Bessenay et al. 2024). These new findings not only extend our understanding of viral replication but also raise several important questions: the existence of hidden layers of temporal gene regulation; the intricate transcriptional interactions among viruses, virophages, and transpovirons; the phenomenon of transient parasitism; and the broader ecological significance of these interactions.

An intriguing hypothesis regarding the formation of chimeric virus–host RNA polymerase complexes – composed of both viral and host subunits – also warrants further investigation, particularly in terms of protein composition and the cellular localization of viral and host components during infection (Bessenay et al. 2024). Further research is needed to assess how widespread this phenomenon is among giant viruses and to quantify the impact of environmental factors on gene expression.

Altogether, these findings emphasize the complexity of transcriptional regulation in giant viruses and their remarkable ability to manipulate host processes during infection. They also point to a promising field for future research, particularly through transcriptomic profiling and single-cell methods (Moniruzzaman et al. 2018; Ku et al. 2020; Bessenay et al. 2024). Continued investigation is likely to reveal new regulatory mechanisms and gene functions that shape the ecological roles of giant viruses across diverse environments.

This review underscores the significance of exploring the molecular architecture of viral genomes and proteomes. Such studies are crucial for understanding the origins, evolutionary mechanisms, and intricate virus–host relationships of giant viruses. Furthermore, these investigations have the potential to uncover novel viral functions and infection strategies, which could have important biotechnological applications. As microbial metagenome databases continue to expand, sequencing, comparative genomics, and phylogenetic analyses of giant virus genomes are essential for accurately identifying viral sequences. These efforts are key to delineating the boundaries between different orders and families of giant viruses and provide a foundation for understanding their evolutionary relationships and taxonomic classification.
